# The impact of age on the Na:K ratio: observations from a general canine population

**DOI:** 10.3389/fvets.2025.1629328

**Published:** 2025-10-24

**Authors:** Polina Zemko, Federico Bonsembiante, Marco Canevelli, Tommaso Banzato

**Affiliations:** ^1^Department of Animal Medicine, Productions and Health, University of Padua, Legnaro, Italy; ^2^Department of Human Neurosciences, Sapienza University, Rome, Italy

**Keywords:** clinical pathology, aging, Addison disease, canine, sodium, potassium

## Abstract

**Introduction:**

The sodium-to-potassium (Na:K) ratio is commonly used as a screening criterion for hyponatremic and/or hyperkalemic hypoadrenocorticism (HA), a serious endocrine disorder in dogs characterized by non-specific clinical signs and variable laboratory findings. A Na:K ratio below 27 typically prompts further investigation through adrenal function tests. However, previous studies suggest that serum K levels may increase with age, even in otherwise healthy dogs. The objective of this study is to evaluate the influence of age on the Na:K ratio, in order to determine whether age-related changes could impact the reliability of this ratio as a screening tool in adult and senior dogs.

**Materials and methods:**

We analyzed biochemical and hematological data from 208 dogs, aged 5–16 years, enrolled in a longitudinal research project of general canine population. The data included their medical history before the control visit and during the 12-month follow-up period.

**Results:**

The prevalence of dogs with a Na:K ratio ≤ 27 was found to be 2.4 ± 2.7% in dogs under 10 years and 12.8±7.0% in those over 10 years. None of the dogs with Na:K ratio ≤ 27 had clinical suspicion of HA, either at the time of initial evaluation or during the 6-months follow-up period. Serum K levels showed a modest but statistically significant age-related increase of 0.22 ± 0.05 mEq/L every 5 years, while Na levels remained stable. As a result, the Na:K ratio declined by 1.5 ± 0.3 points every 5 years. Serum K was moderately correlated with the plateletcrit (PCT) (*r* = 0.39, *p*-value < 0.00001) and PCT was found to increase by 5.9% ± 1.6% every 5 years. It was estimated that each 10% increase in PCT corresponded to 0.142 ± 0.027 mEq/L rise in serum K.

**Discussion:**

The prevalence of dogs with a Na:K ratio ≤ 27 increases with age, reducing the specificity of this threshold for diagnosing HA in older dogs—particularly when Na levels are within the normal range. This decline is due largely, though not exclusively, to age-related increases in PCT, as platelets release K during clotting.

## 1 Introduction

Canine hypoadrenocorticism (HA) is an uncommon endocrine disorder resulting from inadequate production of mineralocorticoids and/or glucocorticoids by the adrenal glands. Although potentially life-threatening, the clinical signs of HA are often vague and nonspecific, including anorexia, lethargy, and sudden gastrointestinal disturbances of varying severity (1, 2). Several subtypes of HA have been identified, each characterized by distinct pathophysiological mechanisms and clinical presentations (1, 3). Diagnosis primarily relies on evaluating adrenal function via the adrenocorticotropic hormone (ACTH) stimulation test, as dogs with HA exhibit a diminished cortisol response to ACTH (4).

Numerous studies have examined clinical and laboratory indicators suggestive of HA (2, 5, 6) among which the sodium-to-potassium (Na:K) ratio is one of the most commonly employed screening tools for mineralocorticoid deficiency. Aldosterone promotes Na reabsorption and K excretion in the distal renal tubules; thus its deficiency leads to hyponatremia and hyperkalemia. A Na:K ratio ≤ 27 has demonstrated a specificity ranging from 87% to 99% for the diagnosis of primary hyponatremic and/or hyperkalemic HA, which is characterized by the destruction of the adrenal gland's zona glomerulosa (5, 6). As a result, adrenal function tests are typically recommended in dogs with a Na:K ratio ≤ 27.

However, a decreased Na:K ratio is not pathognomonic for HA. Several other conditions—including urogenital, cardiorespiratory, and gastrointestinal disorders—can also lead to altered Na:K ratios (3, 7). Moreover, various hematologic and biochemical parameters have been shown to vary with age, even in clinically healthy dogs. Notably, several studies have shown that age-related increase in serum K may mimic the electrolyte abnormalities commonly associated with HA (8, 9).

The objective of this study is to assess the potential influence of age on Na, K and their ratio. We hypothesize that serum K concentration increases with age, potentially rendering the conventional Na:K ratio cut-off of 27 less specific in older dogs. The results presented here are data-driven findings obtained from the analysis of a large dataset of aging biomarkers, compiled as part of a broader study investigating the canine aging process.

## 2 Materials and methods

The data presented in this study were collected as part of a longitudinal project aimed at validating biomarkers of aging in dogs (PROGETTO DI RICERCA DI RILEVANTE INTERESSE NAZIONALE (PRIN) 20228NKPNH—OLD DOG: Validating the dog as an animal model for human aging studies). For each dog, a detailed owner-reported clinical history was obtained, along with documentation of any medical treatment administered during the six months preceding blood sampling. The longitudinal design of the study enabled the systematic collection of follow-up data over time (see Table 1 for an overview of data availability at each time point).

**Table 1:** Types of data available at each time-point of the study.

**Type of data**	**T_0_ time point**	**6-months mark**	**12-months mark**
Clinical history, medical treatment records	208	199	181
Dead/alive status	208	207	202
Standard biochemistry	208	198	181
Standard hematology	207	198	181
Died in the last 6 months^*^	0	6	11

^*^See Supplementary Table 1 for the available information on causes of death.

### 2.1 Population

A total of 208 dogs were enrolled in the study primarily through mailing recruitment. The inclusion criteria were: age over 5 years and absence of clinical signs indicative of a recently developed disorder (e.g., weakness, loss of appetite, fever, acute frequent vomiting, or acute diarrhea). Dogs with unknown birth month and conditions that could interfere with serum sample collection (e.g., persistent lipemia) were excluded. These minimal criteria were chosen to assemble a cohort that broadly reflects the general dog population while minimizing selection bias. Dogs with stable, chronic conditions such as osteoarthritis, degenerative valve disease, pharmacologically controlled endocrine disorders (e.g., hypothyroidism, diabetes mellitus), or history of mild recurrent gastrointestinal disorders were eligible for inclusion. The cohort consisted of dogs of both genders and different neuter statuses (see Table 2 for more details). The breeds most represented in the cohort were the following: Labrador Retriever (15), Golden Retriever (9), Australian Shepherd (8), German Shepard (7), American Staffordshire Terrier (7), Maltese (5), Jack Russel (5), and sighthound-type breeds, including Whippet (5) and Greyhound (4). There were no dogs belonging to Japanese (for example, Akita-inu, Shiba-inu) or Korean breeds or their crossbreeds in the cohort.

**Table 2:** Baseline demographics of the dogs included in the study.

**Age (years)**	**9 (5–16)**
Weight (kg)	21 (2.7 - 80)
Female	122
Male	86
Pure breed	150
Mixed breed	58
Male intact	42
Male castrated	44
Female intact	15
Female spayed	107

For the age and weight, the median values and the range are reported.

### 2.2 Anamnesis

Before the blood test, owners completed a comprehensive questionnaire including information on symptoms observed during the six months preceding the control visit. Although the questionnaire was not specifically designed for the current hypothesis, it covers a wide range of aspects, some of which are relevant and have been selected for analysis in the context of this study. This includes questions on:

- Episodes of vomiting: never/once a month/several times a month/several times a week.
- Episodes of diarrhea: never/once a month/several times a month/several times a week.
- Diagnosed/owner reported gastrointestinal problems.
- Diagnosed endocrine diseases.

### 2.3 Physical examination

For every dog the weight, height, Body Condition Score (BCS) and Muscle Condition Score (MCS^1^) were recorded. General health status was assessed through a standardized physical examination, which also included evaluation of hydration (via skin turgor test), mucous membrane color, and capillary refill time.

### 2.4 Blood sampling

Blood sampling was primarily performed using a closed vacuum system with a butterfly needle and vacutainer tubes (Multifly-Needle 21G, Sarstedt AG & Co KG, Germany), drawing from either the saphenous or cephalic veins. In smaller subjects, however, samples were collected from the jugular vein using a syringe.

Two blood samples were taken from each dog in a consistent sequence to reduce the risk of clotting: the first sample was placed in a K3-EDTA tube for hematology and plasma collection (S-Monovette EDTA K3E 1.2 mL, Sarstedt AG & Co KG, Germany), and the second was collected in a plain tube with clot activator for obtaining serum for biochemical analysis (S-Monovette Serum CAT 4.9 mL, Sarstedt AG & Co KG, Germany). Since the order of sample collection can influence K measurements due to potential contamination from the K3-EDTA anticoagulant, specific precautions were taken to reduce this risk. When sampling with a syringe, the cap of the K3-EDTA tube was removed, and the tube was filled carefully, avoiding direct contact between the syringe and the tube. Whenever possible, the closed vacuum system was used, as it has been shown to reduce the risk of cross-contamination (10–13). All blood samples were collected by the same veterinarian.

The serum tubes were allowed to clot for 10 minutes at room temperature and then centrifuged at 3,000 rpm for 10 min. Biochemical analyses were performed using the BT3500 automatic liquid biochemical analyzer (Futurlab s.p.a., Italy). Complete blood cell count analyses were performed using the Advia 120 automated hematology analyzer (Siemens Healthcare Diagnostic Inc, Germany) with instrument settings and software specifically designed for canine samples while the peripheral blood smear evaluation was performed for each sample by a board-certified clinical pathologist. Samples in which moderate to marked platelets clumps were identified during blood smear examination were excluded from analyses involving platelet count measurements.

Basal serum cortisol concentrations were retrospectively measured for the dogs with altered Na:K ratio in samples stored at -20°C using fluorescence immunoassay analyzer AIA360 VET (Tosoh Bioscience Inc).

### 2.5 Statistical analysis

The statistical analysis was carried out in sequential phases. First, the prevalence of dogs with a Na:K ratio ≤ 27 was calculated using 2-year age intervals, followed by broader age groupings to assess the statistical significance of its age-related change. Consequently, linear regression models were constructed using serum Na, K, and the Na:K ratio as dependent variables, with age as independent variable. The potential influence of additional factors was assessed by sequentially incorporating covariates into the linear model (lm() function in R (14)).

Since K was identified as the primary contributor to the age-related decline in the Na:K ratio, the factors known to elevate serum K levels, including thrombocytosis, marked leukocytosis, hemolysis, acute or chronic renal failure, urinary obstruction, gastrointestinal disorders, and medical treatments were investigated (3, 15–17). Unfortunately, blood gas analysis was not performed in this study, preventing direct assessment of metabolic acidosis, another potential cause of hyperkalemia. Therefore, the potential effects of the following parameters were evaluated:

- Plateletcrit (PCT)
- Leukocyte count
- Hemolysis index
- Serum creatinine
- Azotemia
- Frequency of episodes of diarrhea
- Hydration status
- Medical treatments history

PCT, derived from both platelet count and mean platelet volume, was utilized instead of platelet count alone. This decision stemmed from acknowledging that platelet volume may differ in conditions such as thrombocytopenia recovery and in naturally occurring breed-specific characteristics, like inherited macrothrombocytopenia in Cavalier King Charles Spaniels, which is asymptomatic (18). The authors argue that, because K release during clotting is more associated with overall platelet mass rather than just platelet count, PCT might serve as a more precise indicator in this scenario (19, 20).

To assess the potential impact of gastrointestinal diseases, we identified dogs that experienced diarrhea episodes at least several times per week, as this condition can lead to metabolic acidosis, which may contribute to hyperkalemia. We did not anticipate observing metabolic acidosis in dogs with less frequent diarrhea (less than once a week). To assess the effect of dehydration—another potential cause of metabolic acidosis that may lead to hyperkalemia—dogs exhibiting at least mild dehydration, as determined by the skin turgor test, together with alteration of the capillary refill time and/or mild increase of hematocrit (HCT) or total proteins were identified. Ultimately, the medical treatment history was reviewed to identify dogs that had received, within the 6 months preceding blood sampling, medications known to potentially increase K levels, such as potassium-sparing diuretics, trimethoprim, angiotensin-converting enzyme inhibitors, mannitol, or heparin (4).

Pearson correlation coefficients were calculated between serum K concentration and PCT, leukocyte count, serum creatinine, and azotemia. Parameters showing statistically significant correlations (*p*-value < 0.05) were then included in a linear regression model, with K concentration as the dependent variable.

To explore the differences in the age-related variation of Na:K ratio in dogs with different sex and neuter status we used the analysis of covariance (ANCOVA, anova_test() function in R (14)).

All the statistical analysis was performed with R version 4.3.3 (14).

## 3 Results

**Figure 1:**
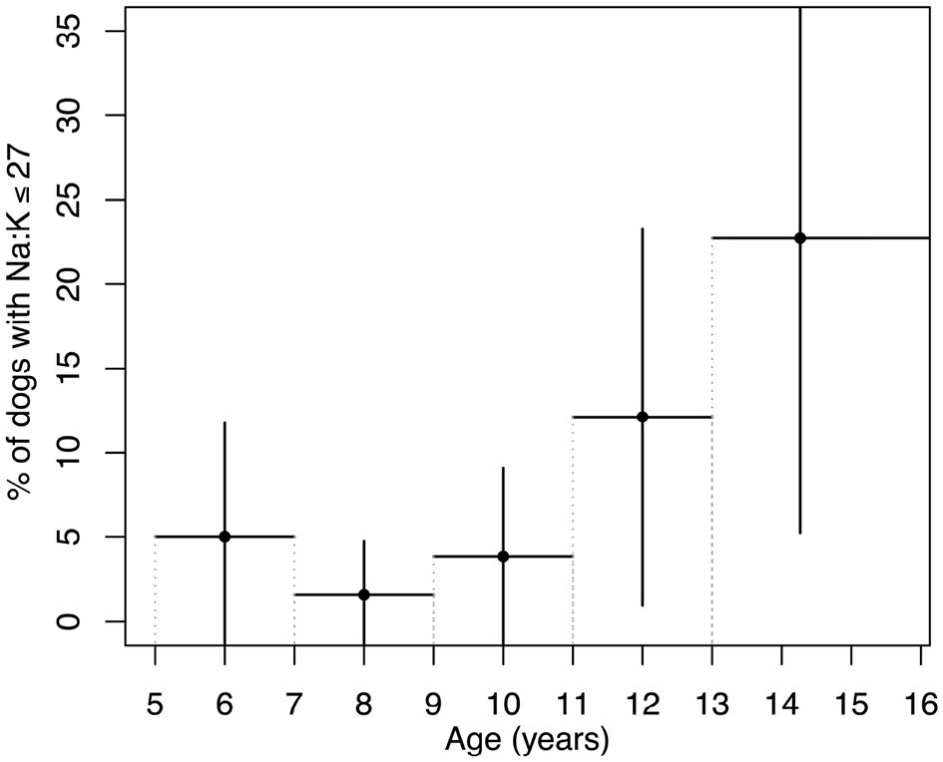
Prevalence of dogs with a Na:K ratio ≤ 27 across age groups. Age groups were defined in 2-year intervals from 5 to 13 years, with all dogs over 13 years combined into a single group to increase sample size. Vertical lines represent the margin of error calculated based on each group's sample size and a 95% confidence interval. Horizontal lines indicate the age range for each group.

14 out of 208 dogs had Na:K ratio ≤ 27. The variation in the prevalence of dogs with a Na:K ratio ≤ 27 across age groups, calculated in 2-year intervals, is shown in Figure 1. Dogs older than 13 years were grouped together to increase the sample size. While the figure suggests a possible increase in prevalence with age, the limited number of dogs in each age group prevented statistical evaluation of the trend. To address this, the cohort was divided into two broader age categories: dogs younger than 10 years and those 10 years and older. The cut-off at 10 years was chosen based on the upward trend observed in Figure 1. In dogs under 10 years of age, the prevalence of a Na:K ratio ≤ 27 was 2.4 ± 2.7%, compared to 12.8±7.0% in dogs over 10 years, confirming a statistically significant difference between the age groups.

The dogs with Na:K ratio ≤ 27 belonged to the following breeds: Pug (3), Shih Tzu (2), Labrador Retriever (2), Basset Hound (1), American Staffordshire Terrier (1), Cocker Spaniel (1), Maltese (1), and Boston Terrier (1). None of these breeds are known to have K-rich RBC due to the activity of the Na-K pump (18). Two dogs with Na:K ratio ≤ 27 were mixed-breed.

The medical histories and follow-up records of the fourteen dogs with a Na:K ratio ≤ 27 were reviewed to evaluate to assess whether this alteration could be attributed to hyponatremic and/or hyperkalemic HA (see Table 3). None of the dogs were clinically suspected of having, nor received a diagnosis of HA either at the time of initial evaluation or during the 12-month follow-up period. However, one dog died before the 6-month follow-up due to suspected neoplasia, and two dogs were lost to follow-up between the 6- and 12-month evaluations. One of these had been diagnosed with Cushing's syndrome and showed bilateral adrenal gland enlargement by the 6-month mark (see Table 3). The other was a 14-year-old dog with pre-existing cardiopathy (MMVD stage B1; (21)) and osteoarthritis, with no other significant health issues reported. Although one dog experienced diarrhea several times per week at the time of the first control and one frequent vomiting, no dogs showed persistent gastrointestinal symptoms in the follow-up.

**Table 3:** Data on the dogs with Na:K ratio ≤ 27.

**Na:K**			**Age**	**Serum**	**Diarrhea**			**Vomiting**			**Health**
**T**_0_	**T**_1_	**T**_2_	**(yr)**	**Cortisol [**μ**g/dL]**	**T**_0_	**T**_1_	**T**_2_	**T**_0_	**T**_1_	**T**_2_	**Issues**
23.9	29.1	30.7	5.1	1.22	0	0	0	2	0	0	NRI
23.9	NA	LFF	12.5	8.35	0	NA	NA	3	NA	NA	Cushing's sindrome diagnosed
24.9	27.1	25.6	10.8	2.66	0	0	0	1	0	0	Recurrent otitis externa
25.2	24.8	LFF	14.0	2.45	0	0	NA	0	0	NA	MMVD B1, osteoarthritis
25.3	27.8	27.0	12.9	0.74	0	0	0	0	0	0	Hypothyroidism
25.4	25.8	23.4	12.5	2.01	0	0	0	0	0	0	NRI
25.5	30.7	NA	14.1	6.31	1	0	0	2	0	0	NRI
25.6	Died	Died	13.1	5.99	0			0			NRI. Death after 2 months for
25.8	31.6	31.2	8.8	0.71	1	1	0	0	0	1	NRI
25.9	NA	NA	14.6	3.27	1	NA	NA	0	NA	NA	Recurrent dermatitis
26.4	28.3	27.0	6.3	5.90	0	0	0	0	0	0	Idiopathic epilepsy
26.7	26.5	28.0	13.9	1.39	3	0	0	0	0	2	Recurrent otitis externa
26.9	28.6	29.0	10.4	2.01	0	0	0	0	0	0	NRI
27.0	29.5	29.1	11.1	2.40	0	0	0	2	2	1	Hypothyroidism

The columns T_0_, T_1_, and T_2_ refer to data collected at the initial evaluation, 6-month follow-up, and 12-month follow-up, respectively. Vomiting and diarrhea episodes are rated on the following scale: 0 = never, 1 = once a month, 2 = several times a month, 3 = several times a week. NRI, No Reported Issues; LFF, Lost to Follow-up; NA, Data not available, although the dog continued to participate in the study.

To ulteriorly explore the possibility of HA in the dogs with Na:K ratio ≤ 27 the basal serum cortisol levels were retrospectively measured in their stored serum samples (see Table 3). All of them had basal serum cortisol levels above the cut-off value of 0.58 μg/dL (22).

**Figure 2:**
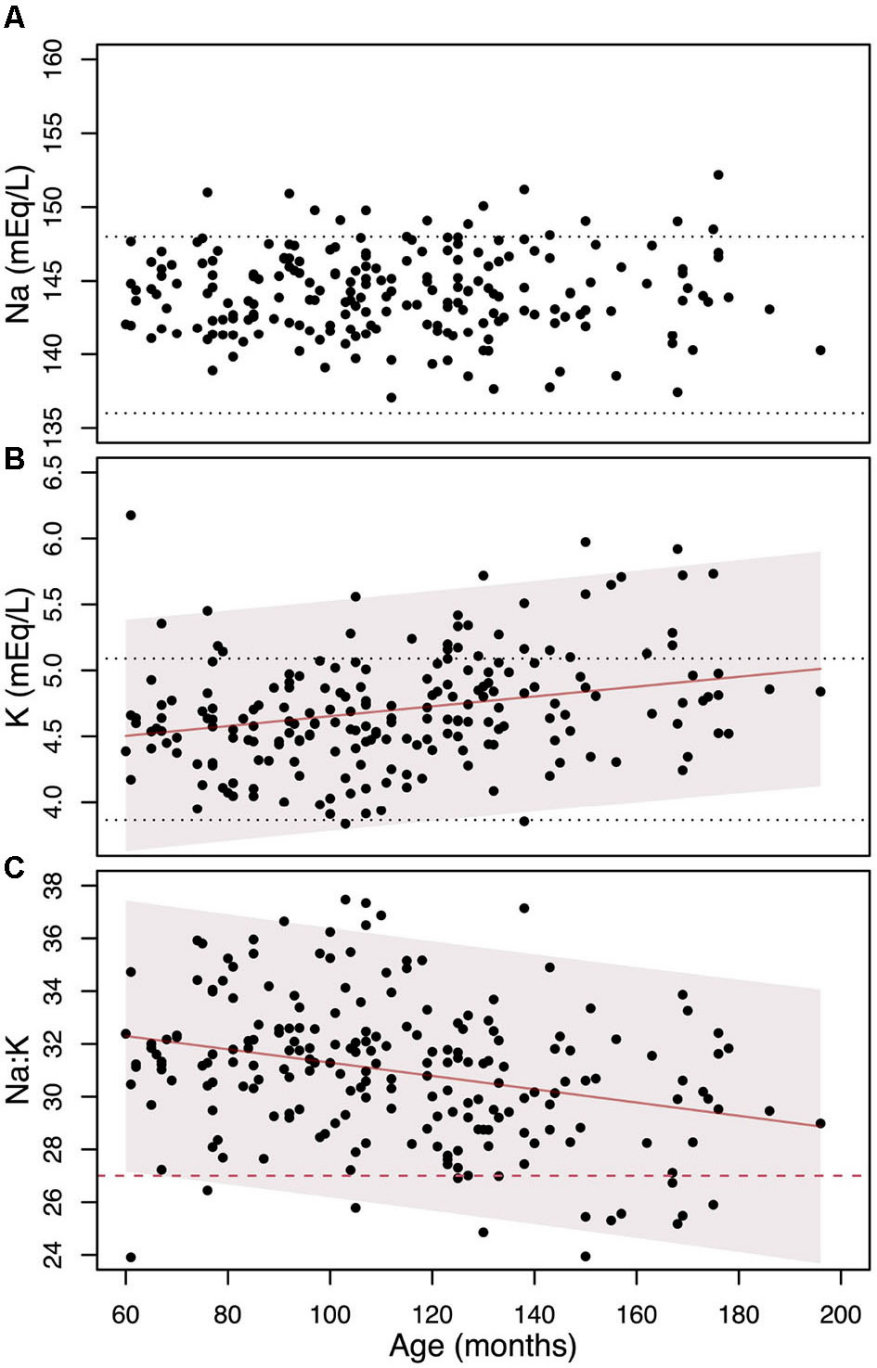
The change in Na **(A)**, K **(B)**, and their ratio **(C)** as individuals age, along with the linear regression models and their 95% confidence intervals (represented by shaded areas). The black dotted horizontal lines denote the laboratory reference values. The red dashed line on the **(C)** indicates the Na:K ratio cut-off level of 27.

To investigate the cause of the increase in the prevalence of dogs with a Na:K ratio ≤ 27 with age, we analyzed age-related variations in Na, K, and the Na:K ratio, as presented in Figure 2. Serum Na concentrations showed no significant age-related changes (β = -0.006±0.010, *p* = 0.55). While 15 dogs had Na levels above the laboratory reference range, none were hyponatremic. In contrast, serum K levels showed a small but statistically significant increase with age (β = 0.0037 ± 0.0009, *p* = 0.0002, R^2^ = 0.059). 32 dogs had serum K levels above the upper laboratory limit and the percentage of dogs with K above the reference range increases with age: 7.4% (9 out of 122) in dogs aged 5 to 10 years and 27% (23 out of 86) in those over 10 years. As a result, the Na:K ratio demonstrated a reliable age-related decline of -1.5±0.3 points every 5 years (β = -0.025±0.005, *p* = 0.00002, R^2^ = 0.079).

When weight was added as additional predictor to the linear model (Na:K ~ age), its contribution was not statistically significant (*p* = 0.41). No significant differences were observed in the Na:K ~ age relationship across groups defined by sex (*p* = 0.47) or neuter status (*p* = 0.28).

Since the age-related variation in the Na:K ratio was found to be solely attributed to changes in K levels, we have focused on identifying the factors that influence serum K concentration.

Across the entire cohort, only one dog—which had a documented heart murmur but had never undergone echocardiographic evaluation (Na:K ratio = 32, K=4.52 mEq/L)—received treatment with both benazepril and spironolactone prior to blood collection. Four dogs in the cohort were reported to have recurrent diarrhea (K=4.39, K=4.40, K=4.61, and K=5.28 mEq/L, respectively) and one was classified as mildly dehydrated based on the skin turgor test and showed increased HCT and total proteins being otherwise completely asymptomatic (K = 4.72 mEq/L). Excluding these six dogs from the calculations did not substantially affect the results: the prevalence of dogs with altered Na:K ratios remained nearly unchanged (2.5 ± 2.7% for dogs <10 years and to 12.3 ± 7.1% for dogs >10 years), the association between K and age remained the same (β = 0.0037 ± 0.0009, *p* = 0.0002, R^2^ = 0.057). These findings indicate that the presence of these dogs cannot account for the observed trends.

After excluding dehydration, medical treatments, and recurrent diarrhea as potential explanations for the observed variation of K with age, we focused on other possible factors described in the ‘Statistical analysis' subsection. The six dogs identified above were excluded from the cohort to minimize their contribution, and Pearson correlation coefficients between K levels and the investigated factors were then calculated for the remaining 202 dogs (Table 4). As shown, the only factor influencing K levels, aside from age, is PCT.

**Table 4:** Correlation between the serum K concentration and the following parameters.

**Variable**	**Correlation**		**Linear regression model**		
	*r*	*p* **-value**	β	*R* ^2^	*p* **-value**
Age (months)	0.25	0.00034	0.0037 ± 0.001	0.057	0.0003
PCT %	0.39	<0.00001	0.0155 ± 0.0026	0.15	<0.00001
Leukocyte count × 10^3^ cells μL	0.046	0.52			
Hemolysis index	-0.002	0.97			
Creatinine mg/dL	-0.10	0.15			
Azotemia mg/dL	0.14	0.04			

Pearson linear coefficient *r* is reported together with the corresponding *p*-value. The last 3 columns represent the regression linear model coefficients with K as dependent variable.

Adding PCT as an additional variable to the linear regression model (K ~ age) raised the adjusted R^2^ from 0.059 to 0.17, indicating that PCT accounts for a substantial portion of the variation in K. In our dataset, PCT also shows a statistically significant increase with age (β = 0.099 ± 0.027, *p* = 0.0003, R^2^ = 0.061), underscoring the importance of including both PCT and age in the model to distinguish their individual contributions to serum K levels. The parameters of the final linear model (K ~ PCT + Age) are presented in Table 5.

**Table 5:** Parameters of the multiple linear regression model: K ~ PCT + Age (months).

	**β**	***p*-value**	**Adjusted R^2^**
PCT	0.0142 ± 0.0027	<0.00001	
Age (months)	0.0021 ± 0.0010	0.049	
Model		<0.00001	0.17

Regression coefficients (β) with the corresponding standard errors and *p*-values for each variable are presented together with the *p*-value and the adjusted *R*^2^ of the model.

**Figure 3:**
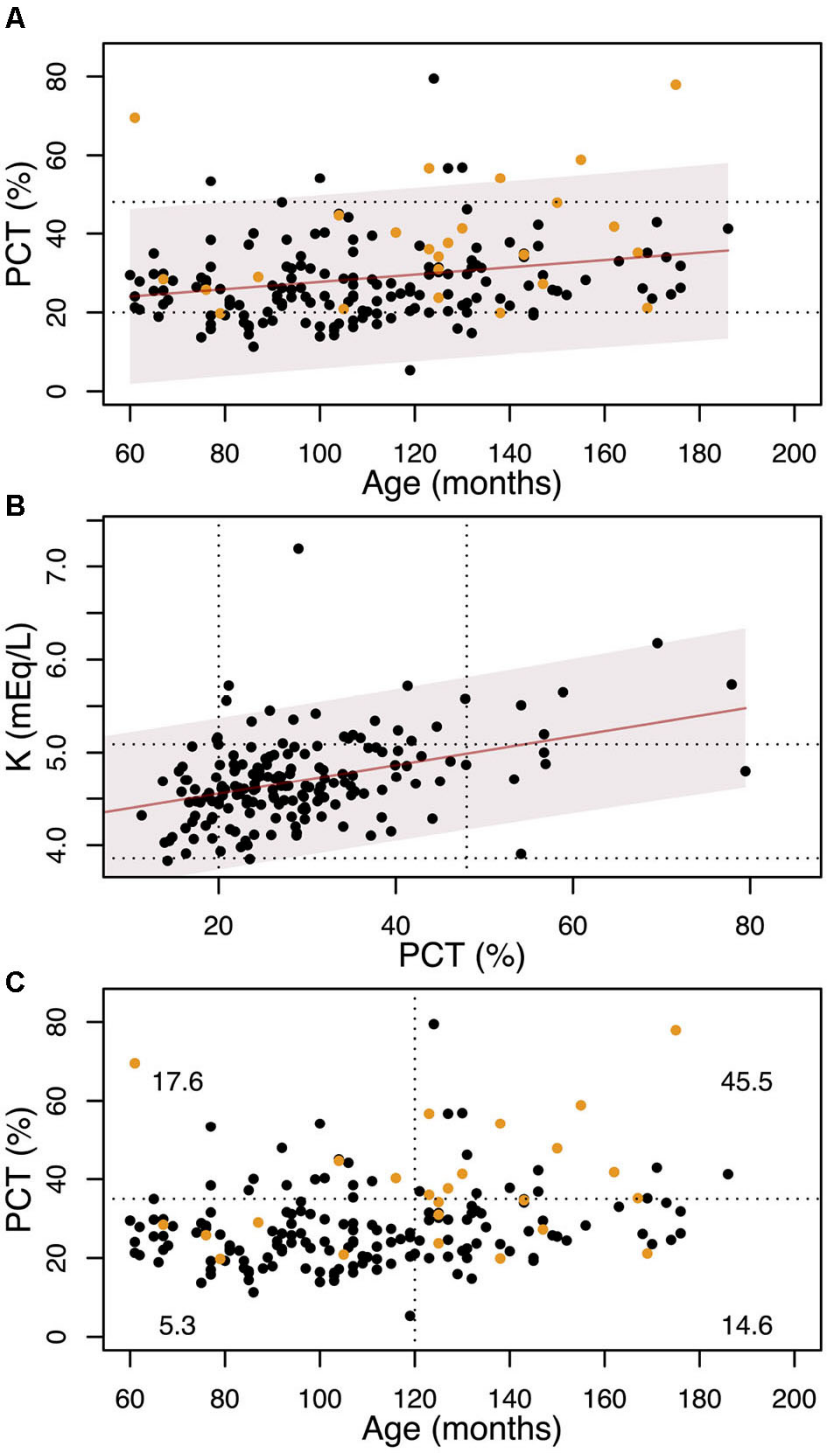
**(A)** variation with age of the PCT. The solid line shows the linear regression, and the shadowed region is the 95% confidence interval. The dotted lines show the laboratory range for the PCT. Yellow dots represent dogs with serum K concentration above the laboratory interval (5.09 mEq/L). **(B)** variation of the K concentration with PCT. The solid line shows the linear regression and the shadowed region is the 95% confidence interval. The dotted lines show the laboratory ranges for the PCT and K. **(C)** The dotted lines show the 10 years cut-off and the PCT=35% cut-off. Yellow dots represent dogs with serum K concentration above the laboratory interval (5.09 mEq/L). At each quadrant we report the percentage of dogs with K≥5.09 mEq/L.

In Figure 3, the relationship between PCT and age is reported, as well as the association between serum K concentration and PCT. Figure 3C shows the percentage of dogs with Na:K ≤ 27 in function of age and PCT. It can be seen that almost 46% (10 out of 22) of the dogs over 10 years old with PCT>35% (noting that the upper reference limit is 48%) have serum K levels exceeding the reference interval.

**Figure 4:**
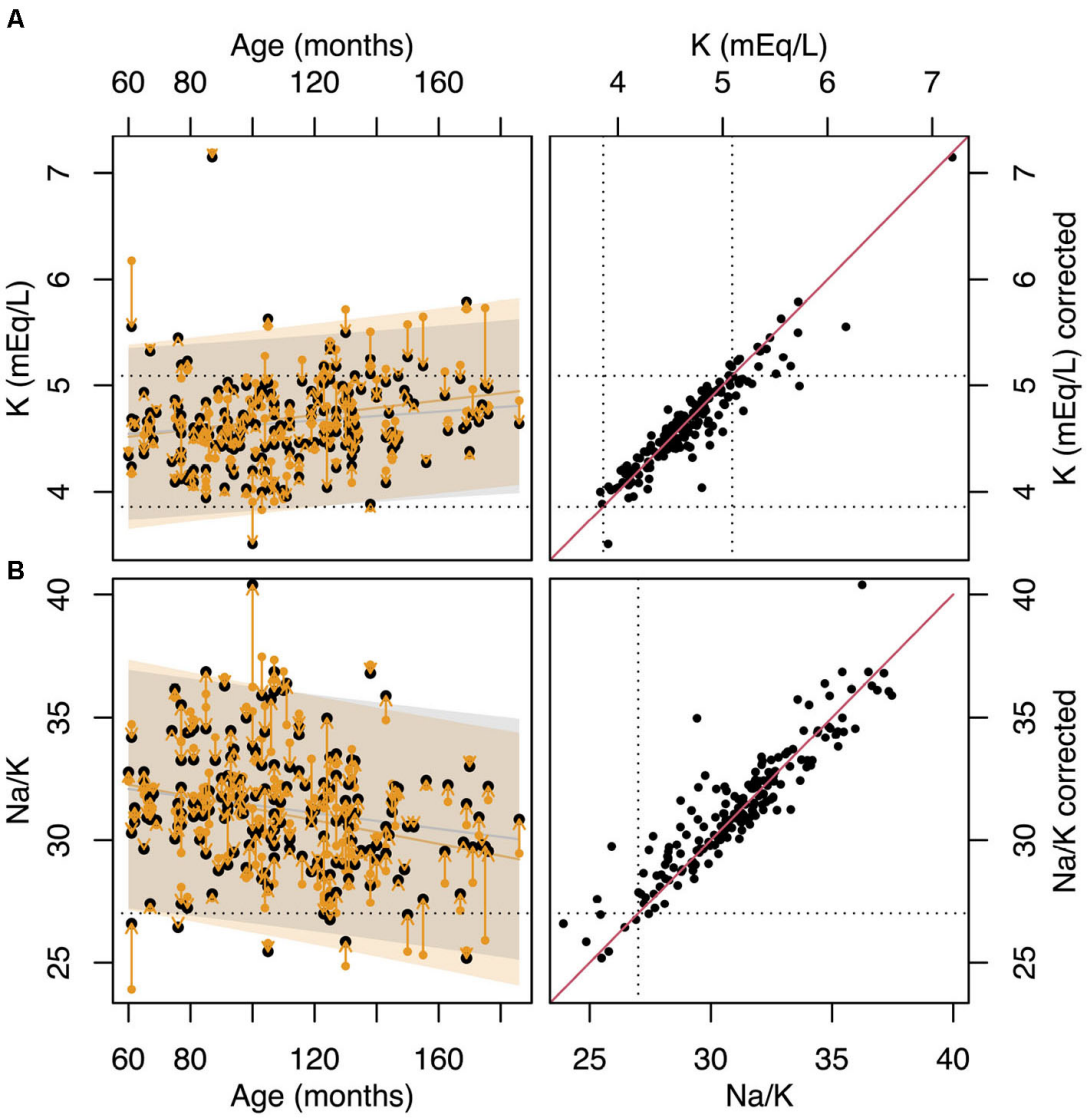
**(A)** variation with age of K. Yellow points show the original data while the black points show the values of K normalized to PCT=26%. The dotted horizontal lines represent the laboratory reference range for K. **(B)** variation of the Na:K ratio with age. Yellow points show the original data, while the black points show the values of Na:K calculated using K normalized to PCT=26%. The dotted horizontal line shows the Na:K=27 cut-off limit. **(C)** values of K vs values of K normalized to PCT=26%. The dotted horizontal lines represent the laboratory reference range for K. The solid red line represents the line of identity. **(D)** values of Na:K ratio vs values of Na:K ratio calculated using the K normalized to PCT=26%. The dotted horizontal lines represent Na:K=27 cut-off limit. The solid red line represents the line of identity.

To account for K released from platelets, serum K levels can be adjusted based on each dog's individual PCT value. Figure 4 shows the variation of K and the Na:K ratio with age using both the original data and K levels normalized to a PCT value of 26%. This normalization point was arbitrarily selected as it represents the median PCT value in our cohort. The correction reveals that, in several cases—particularly in older dogs—both K levels and the Na:K ratio shift back into the reference range after adjustment.

However, as indicated by the linear model in Table 5, increased PCT is not the only factor contributing to the variation of in serum K levels and the Na:K ratio. Even after adjusting for the effect of PCT, a significant residual association between age and the Na:K ratio persists (β = -0.016, *p* = 0.009, R^2^ = 0.032).

## 4 Discussion

The results demonstrate a presence of statistically significant increase of the prevalence of dogs with Na:K ratio ≤ 27 with age. While the diagnosis of HA requires confirmation via an ACTH stimulation test, such testing was beyond the scope of the present study. However, considering the low estimated prevalence of HA in dogs (0.06–0.28%, (23–25)), the lower median age at diagnosis comparing to the median age of our cohort, and the absence of a diagnosis or persistent symptoms suggestive of HA in any of the dogs with a Na:K ratio ≤ 27—either previously or during the 12-month follow-up period—we consider this possibility to be extremely unlikely. Moreover, the basal serum cortisol concentrations all dogs with a Na:K ratio ≤ 27 were above the threshold of 0.58 μg/dL, a value previously shown to yield 100% sensitivity and 97% specificity for the diagnosis of hyponatremic and/or hyperkalemic HA (22).

The increasing prevalence of dogs with a Na:K ratio ≤ 27 with age suggests that the Na:K ratio should be interpreted with caution in older dogs, particularly those with normal Na levels. Although HA is most commonly diagnosed in adult dogs—with a median age at diagnosis of 3–4 years—it can still occur in dogs as old as 14 years (1). Incorporating age into the calculation of Na:K ratio cut-off values in dogs may improve the specificity of this diagnostic criterion. However, considering that the Na:K ratio is primarily employed as a screening test, sensitivity continues to outweigh specificity in importance.

The observed increase in the prevalence of dogs with a Na:K ratio ≤ 27 is likely due to the age-related rise in serum K concentration, as previously reported (8, 9). This increase may be attributed to several factors, including contributions from the blood clotting process, leukocytosis, impaired renal function, metabolic acidosis, and gastrointestinal disease. As our cohort consisted exclusively of dogs without signs of acute disease, and none were pregnant, several potential causes of elevated K—such as chronic effusions with repeated drainage, pregnancy, congestive heart failure, rhabdomyolysis, or other forms of muscle damage—were effectively excluded (3, 17). In our cohort of 208 dogs, only one dog was treated with drugs known to potentially affect serum K levels.

Our analysis revealed a substantial contribution of the coagulation process to serum K levels: incorporating PCT into the linear regression model allows us to explain another 11% of the variation in serum K concentration. Specifically, a 10% increase in PCT is associated with a 0.14 mEq/L increase in serum K—a substantial change considering that the entire laboratory reference interval for K spans only 0.62 mEq/L. As shown Figure 3B, about half of the dogs with PCT values near the upper reference limit already exhibit serum K concentrations above the normal range, regardless of age.

The platelet counts were reported to increase with age in dogs (9, 26). The same trend was observed in our cohort: the PCT rises with age at roughly 6% every 5 years, leading to an enhanced contribution to the K concentration from the clotting mechanism as one ages. Given that platelet-induced K release can significantly affect the measured K concentration, a potential solution is to measure K in heparinized plasma or through blood gas analysis to avoid the release of K from the platelets. In dogs experiencing an increased platelet count, the K levels can be modified through the correlation between PCT and K mentioned earlier: a 10% rise in PCT equates to a 0.14 mEq/L alteration in K concentration. Blood gas analysis offers the additional advantage of improved accuracy through direct potentiometry, which is particularly beneficial in cases of hypoproteinemia or lipemia, where indirect potentiometry used in standard serum biochemistry may be less reliable.

Nonetheless, despite accounting for the K released from platelets, part of the age-related decrease in the Na:K ratio persists, leading some dogs to have values still below the Na:K threshold of 27. No significant contributions were identified from elevated leukocyte counts or hemolysis. Although leukocytes are rich in intracellular K and can potentially elevate serum K levels if ruptured during blood collection, this is typically associated with the use of narrow-gauge needles, delays in sample processing, or hematologic conditions such as lymphoid or myeloid leukemia, where there is a marked leukocytosis and the leukocytes become more fragile. To minimize such risks, a standardized blood collection protocol was implemented. In our cohort, only one dog was diagnosed with a possible chronic lymphoid leukemia during the six-month follow-up period, and this case did not present leucocytosis.

The effect of hemolysis may be significant in breeds with K^+^-rich red blood cells. However, since our cohort does not include any dogs of Japanese or Korean breeds—known for this trait—the absence of a correlation between the hemolysis index and serum K levels is consistent with expectations.

No correlation was found between K and serum azotemia or creatinine levels. However, interpreting creatinine levels in older dogs can be challenging due to age-related muscle mass loss, leading to reduced serum creatinine concentration, which may mask declining renal function. Several studies have shown that creatinine concentration decreases with age in dogs (8, 9). Notably, among dogs with a Na:K ratio ≤ 27, six out of fourteen had a MCS of 3 or higher, indicating moderate to severe muscle loss (see text footnote 1). Thus, we cannot exclude the contribution of declining renal function to the age-related increase in K concentration.

Unfortunately, we lack data on blood pH, so we cannot rule out metabolic acidosis as a potential cause of elevated K levels in older dogs. Radakovich et al. (9) suggested the possibility of metabolic acidosis induced by dehydration to explain the greater anion gap and the increase in serum K in older dogs. However, only one dog in our cohort showed signs of mild dehydration, limiting our ability to explore this as a contributing factor. Frequent diarrhea, another potential cause of acidosis, was observed in only four dogs—an insufficient number to account for the age-related trend observed.

This study has several limitations. First, ACTH stimulation tests were not performed, preventing definitive exclusion of hyponatremic and/or hyperkalemic HA in the cohort. Second, the absence of blood gas analyses limited our ability to assess whether metabolic acidosis was present in any of the dogs. Additionally, symmetric dimethylarginine (SDMA) levels were not measured; SDMA is a more sensitive and muscle mass-independent marker of renal function and could have provided further insight into the potential age-related decline in kidney function, which may explain the observed gradual increase in serum K levels with age. Unfortunately, the available data on creatinine and azotemia were insufficient to explore this hypothesis.

The findings of this study suggest that a Na:K ratio ≤ 27 should be interpreted with caution in older dogs, particularly those with normal Na levels, as it becomes a less specific marker for Addison's disease in this population. Enhancing diagnostic accuracy could be achieved by assessing K levels in heparinized plasma or through blood gas analysis, thereby preventing the false increase caused by platelet release. Alternatively, serum K values may be corrected for clotting-related contributions using the estimate that each 10% increase in PCT raises serum K by approximately 0.14 mEq/L. Incorporating age and PCT into the calculation of Na:K cut-off values may further improve the specificity of this diagnostic criterion for identifying HA in dogs.

## Data Availability

The original contributions presented in the study are included in the article/Supplementary material, further inquiries can be directed to the corresponding author/s.

## Footnotes

^1^ https://wsava.org/wp-content/uploads/2020/01/Muscle-Condition-Score-Chart-for-Dogs.pdf
