# Supplementary material for: The impact of age on the Na:K ratio: observations from a general canine population
Source: Front Vet Sci. 2025 Oct 24;12:1629328. doi: 10.3389/fvets.2025.1629328 (PMC12593493; doi:10.3389/fvets.2025.1629328)
Supplement: Supplementary file 1 [file Table_1.docx]

|  | Age at death | Cause of death | Symptoms prior to death | Eutanasia (yes/no) |
| --- | --- | --- | --- | --- |
| Died within 6-months | 13.3 | Tumor (suspected) |  | No |
|  | 12.9 | Sudden death |  | No |
|  | 16.5 | Unknown | Disorientation, restlessness, vocalization | Yes |
|  | 14.7 | Thyroid tumor |  |  |
|  | 6.8 | Tumor |  |  |
|  | 13 | Unknown | Epilepsy crisis, acute tetraplegia | Yes |
| Died within 12-months | 9.5 | Tumor |  |  |
|  | 11.5 | Vertebral tumor or spinal disc extrusion | Acute papaplegia | Yes |
|  | 11.1 | Osteosarcoma |  | Yes |
|  | 13.3 | Unknown | Acute diarrhea | No |
|  | 13.8 | Splenic mass lesion |  | Yes |
|  | 15.1 | Unknown |  |  |
|  | 11.1 | Melanoma |  |  |
|  | 6.3 | Osteosarcoma |  | Yes |
|  | 15.4 | Osteolytic mass lesion (C1 vertebra) |  |  |
|  | 13.6 | Metastatic tumor | Multiple hepatic and spleen mass lesions | Yes |
|  | 12.1 | Pericardial effusion |  | Yes |

Supplementary Table1. Available data on the causes of death for dogs that died during the follow-up period.
